# Reinforcement Corrosion Testing in Concrete and Fiber Reinforced Concrete Specimens Exposed to Aggressive External Factors

**DOI:** 10.3390/ma16031174

**Published:** 2023-01-30

**Authors:** Wioletta Raczkiewicz, Magdalena Bacharz, Kamil Bacharz, Michał Teodorczyk

**Affiliations:** 1 Department of Strength of Materials and Building Structures, Faculty of Civil Engineering and Architecture, Kielce University of Technology, 25-314 Kielce, Poland; 2 Independent Researcher, 03-193 Warsaw, Poland

**Keywords:** AE method, chloride corrosion, concrete cover, freeze–thaw cycles, GP method, rebar, steel fibers

## Abstract

One of the leading causes of reinforced concrete degradation is chloride attack. It occurs due to the penetration of chlorides through pores and cracks into the concrete cover. This phenomenon becomes more dangerous if reinforced concrete elements are subjected to cyclic temperature changes. The concrete cover protects against corrosion. This paper presents research, the primary purpose of which was to determine the effect of the addition of steel fibers to concrete on the development of corrosion of the main reinforcement. The tests were carried out on three types of reinforced concrete specimens made of ordinary concrete and concrete with different amounts of steel fibers (0.25% and 0.50%). In order to initiate corrosion processes, specimens were partially submerged in a 3% sodium chloride solution and were subjected to freeze–thaw cycles. The electrochemical polarization galvanostatic pulse method was used for analyzing the reinforcement corrosion activity. Moreover, it was verified whether the corrosion of reinforced concrete elements affects the acoustic emission wave velocity. The addition of steel micro-reinforcement fibers increases the corrosion resistance of reinforced concrete. In addition, a strong linear correlation between the AE wave velocity and the values of the corrosion current density was revealed.

## 1. Introduction

**Figure 1:**
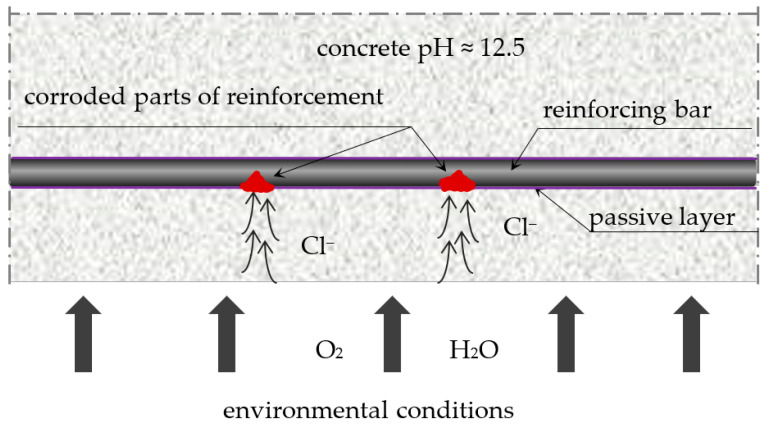
Pitting corrosion in concrete.

Chloride corrosion [1,2] is, apart from carbonation, one of the main causes of reinforcement corrosion in reinforced concrete structures. Reinforced concrete structures of roads and bridges are particularly exposed to this type of corrosion (due to the use of sodium chloride as a surface de-icing agent in winter), as well as facilities located in coastal areas, mainly port facilities, both engineering and industrial (due to the presence of sodium chloride in seawater and in the so-called salt fog in the air). Chloride ions (Cl^−^) penetrate the concrete and move through the liquid-filled capillaries by diffusion into the concrete cover [3,4,5,6,7]. Over time, these ions reach the passive layer protecting the reinforcement and damage it, which initiates the process of electrochemical corrosion of the reinforcement (Figure 1). Depending on the environmental conditions, this process develops at different rates. Ions penetrate deeper and faster into the concrete structure together with the liquid, the more humid the environment is, and the concrete is more porous, with continuous (connected) pores. If there are cracks in the concrete, the progress of ion diffusion is even faster, thereby increasing the risk of corrosion. Any micro-defects and defects in the concrete increase the risk of initiating and developing chloride corrosion. Micro-defects include shrinkage cracks in large-area and large-size elements of roads and bridges. They form due to the intensive evaporation of water from the surface of the elements in the concrete mixture setting and hardening and due to exothermic processes of binding large masses of the mixture (e.g., when making abutment bridges). Stresses resulting from this may lead to numerous micro-cracks of the concrete cover, which reduces its tightness. In addition, frost and repeated freezing and thawing of the liquid in concrete pores can also damage the concrete cover [8,9]. Other defects may arise as a result of mechanical damage related to, for example, overloading of the structure and improper use of engineering facilities.

It is worth adding that, in the case of corrosion caused by chlorides, we are dealing with the so-called pitting corrosion [10,11,12,13]. It is so dangerous that corrosion centers are formed pointwise and can lead to the bursting of the concrete cover from the inside without visible changes on the surface of the element.

The key factor in protecting the reinforcement against corrosion caused by the synergy of chloride ions and frost is a properly designed and made concrete cover, which should be of appropriate thickness, characterized by high tightness, preventing the penetration of ions [14,15] and ensuring frost resistance. For this reason, it is recommended to adopt a low w/c ratio, add an air-entraining agent and use metallurgical cement instead of Portland cement, which, due to the addition of granulated blast furnace slag, makes the concrete tighter [16,17,18,19]. However, concrete with metallurgical cement, although it protects the reinforcement better against chloride corrosion, is less resistant to carbonation caused by the effect of carbon dioxide on concrete. Meanwhile, in real conditions, there is often a synergy of these factors. Therefore, in addition to using different types of cement, other solutions are sought to effectively improve the tightness of concrete. One such solution is improving concrete mixture parameters not by interfering with its chemical composition but by adding randomly dispersed micro-reinforcement fibers. Furthermore, the presence of fibers in structural concrete is recommended due to the reduction of shrinkage deformations and increasing the strength parameters of this material.

Research on concrete with dispersed reinforcement, i.e., fiber-reinforced concrete, has been conducted for decades, and many of its properties have already been recognized and described [20,21,22]. Various fibers can be used as dispersed reinforcement: steel, synthetic (polypropylene, polyester, polyacrylonitrile), glass, carbon, basalt or even organic. The basic parameters characterizing fibers of selected types, helpful in determining their suitability for use, were developed by, among others, the authors of the publication [23].

Fiber-reinforced concrete is a “quasi-plastic” and “quasi-homogeneous” material, having better adhesion, deformability, tightness and higher early strength [21,22,23,24,25,26,27,28]. This is important in the case of structures exposed to aggressive environments (i.e., bridges, tunnels, viaducts, car parks), thin-walled elements (tanks and basins of swimming pools), weirs, retaining walls, elements subjected to dynamic loads and concrete surfaces, including airfield pavements [29,30] and industrial floors. Randomly dispersed fibers in the concrete mix reduce stress concentration and thus limit cracking [20,21,22,28,31]. When added to the fresh concrete mix, they play a micro-reinforcement role, reducing plastic shrinkage. In hardened concrete, fibers limit the formation of cracks from drying [32,33].

Moreover, adding fibers to the concrete mix affects its air entrainment, which improves frost resistance [34]. Randomly dispersed fibers block the formation of a network of interconnected pores in the concrete, making it difficult for various substances to penetrate it. For the above reasons, adding fibers should improve the properties of the concrete cover as a layer protecting the reinforcement and thus mitigate the corrosion of reinforcing bars in reinforced concrete elements.

One of the most commonly used fibers, due to their multiple effectiveness aspects and relatively low price, is steel. These types of fibers are produced in various shapes and sizes. The content of steel fibers in the concrete mixture usually ranges from 0.25 to 2% (by volume) per 1 m^3^ [20,21,22,23]. Adding fiber reinforcement in less than 0.25% is ineffective (the tests show that it does not improve concrete parameters). On the other hand, adding more than 2.0% makes the concrete mix very difficult to work with (even with superplasticizers). The authors’ research shows that mix preparation is challenging even with adding fibers in the amount of 1.5%. Due to the formation of “nests”, i.e., clusters of tangled fibers, the mix becomes heterogeneous, and the concrete parameters are worse than those of mixed with 1.0% fiber addition [35,36].

**Figure 2:**
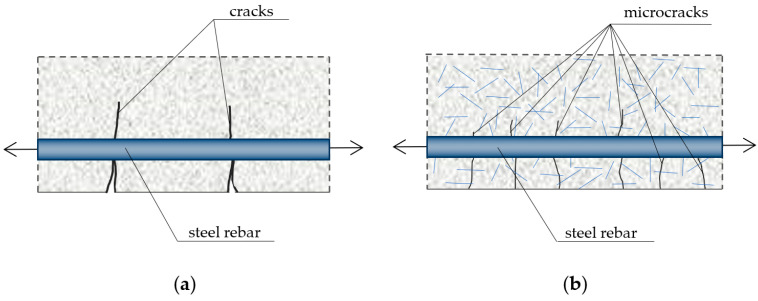
Size and distribution of cracks in reinforced concrete elements subjected to tension: (**a**) ordinary concrete; (**b**) concrete with dispersed reinforcement (based on [21]).

The purpose of adding steel fibers to the concrete is to increase the composite’s strength and mechanical properties, including tensile strength, fatigue strength, impact strength, crack resistance and abrasion resistance [20,21,22,23], as well as to increase its cohesion and homogeneity. Cohesion improvement in concrete containing fibers results from the effect of “fastening” the concrete matrix, preventing the formation of large pores in the concrete mix and limiting the formation and spread of shrinkage cracks [21] during concrete setting and hardening, which is schematically shown in Figure 2.

**Figure 3:**
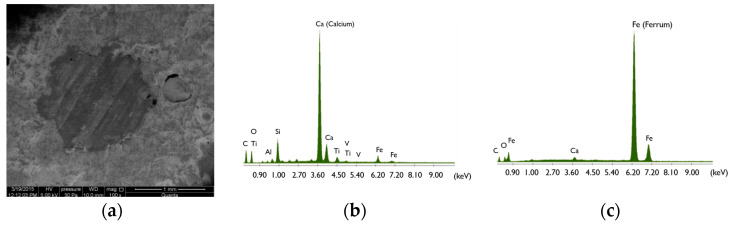
Fiber concrete sample: (**a**) image of the sample in cross-section through the fiber, (**b**) EDS analysis surrounded by the fiber and (**c**) through the fiber.

Some researchers suggest that concretes with dispersed steel micro-reinforcement are not resistant to corrosion [23,24]. Others [22,35] believe that steel fibers do not corrode because they are too short for corrosion centers to form. In addition, the fibers are not connected to each other and are made of more resistant steel. If there are single cases of corrosion, the volume of the fiber after corrosion is so slightly greater that it does not break the concrete and even increases the anchoring force with the matrix. The authors of this paper conducted research in this area, e.g., based on images from a scanning microscope and EDS analysis [36]. Figure 3 shows a scanning microscope image of the concrete sample cut from the concrete cover, with the visible fiber section, as well as the charts from EDS analysis performed for the concrete covering the fiber and the fiber itself. The observed results of the analysis showed no corrosion products in the structure of the concrete around the fiber.

Nevertheless, considering the above discrepancies in the corrosion resistance of fiber-reinforced concrete with dispersed steel reinforcement, it is worth analyzing in laboratory conditions.

This article presents research aimed at evaluating the effect of adding steel fibers to concrete on the development of the corrosion process of ordinary reinforcement in specimens exposed to chloride ions and frost. Two non-destructive methods were used in the tests: the electrochemical polarization galvanostatic pulse method using the GP-5000 GalvaPulse^TM^ measuring set [37] and the acoustic emission method for the comparative analysis of mechanical wave parameters caused by the calibration pulse of acoustic sensors and propagating in concrete and fiber-reinforced concrete due to the action of chloride ions and frost.

## 2. Materials and Methods

### 2.1. Research Material

The components of the concrete mixture and their quantity per 1m^3^ of concrete are listed in Table 1.

**Figure 4:**
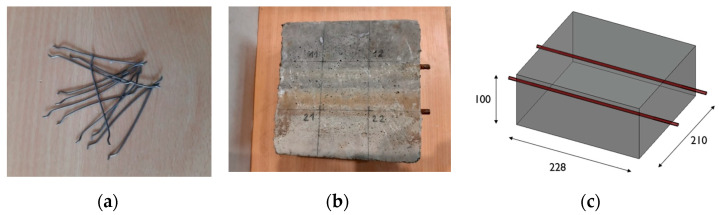
(**a**) Steel fibers used to make the specimens, (**b**) selected test specimen, (**c**) scheme of the tested specimen. Dimensions are given in (mm).

The specimens were made in three series. The first series consisted of “witness specimens” made of the base concrete mixture (marked with the symbol C). The specimens in the two other series were made from a mixture to which randomly dispersed steel fibers were added in various amounts. The specimens with 0.25% fibers by volume of the mixture were marked SF_0.25, and those with 0.5% were marked SF_0.5. In the tests, straight fibers with hooked ends of the BauMix 60/1 type, 60 mm long and 1.0 mm in diameter, were used (Figure 4a).

Six specimens with dimensions of 210 × 228 × 100 mm^3^ were prepared (Figure 4b,c), including two C specimens (witnesses), two SF_0.25 specimens and two SF_0.5 specimens, for the main tests aimed at assessing the corrosion of reinforcement in concrete. In all specimens, two ribbed bars with a diameter of ϕ 8 mm made of BST 500 steel, placed in parallel at 70 mm spacing from the side edges of the specimens, were concreted (Figure 4b). The adopted concrete cover was 25 mm.

Three cubic specimens in each series (C, SF_0.5 and SF_0.25) were also prepared to determine the compressive strength according to [38,39].

All specimens were made under identical laboratory conditions at 20 ± 2 °C and relative humidity 50 ± 5%. The specimens were removed from the molds the next day after concreting. 

**Figure 5:**
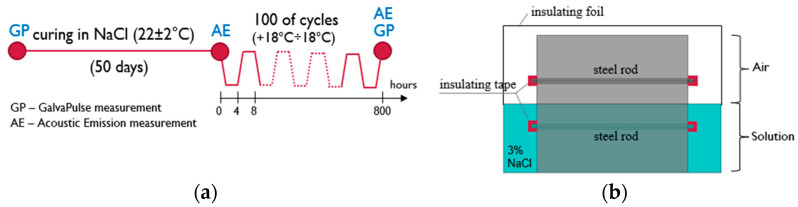
(**a**) The course of the test with marked measurement points; (**b**) test scheme.

In order to reflect the influence of an aggressive corrosive environment and initiate corrosion processes, the specimens were placed in a plastic tub in a 3% sodium chloride solution, which was tightly wrapped with foil. The immersion of the specimens was partial—it covered only half of each specimen with one reinforcement bar, which was to assess the impact of ambient conditions on the activation of the corrosion process. In the immersed parts of the specimens, Cl^−^ ions were transported with the liquid directly into the concrete. In the specimen parts above the surface of the solution, the chloride ion migration was due to capillary action and the action of salt fog. The specimens were immersed for 50 days in a 3% NaCl solution. After this time, the specimens (still immersed in the solution) were placed in a freezing chamber and subjected to 100 cycles of freezing and thawing. Freezing cycles were carried out in a frost resistance test chamber with an automatically controlled research program. The temperature range was +18 ÷ −18 °C. There were ~3 cycles per day. The course of the test is shown in Figure 5a. During the freeze–thaw cycles, reinforcement bar ends protruding from the specimens were insulated. They did not come into contact with the solution, as shown schematically in Figure 5b.

**Figure 6:**
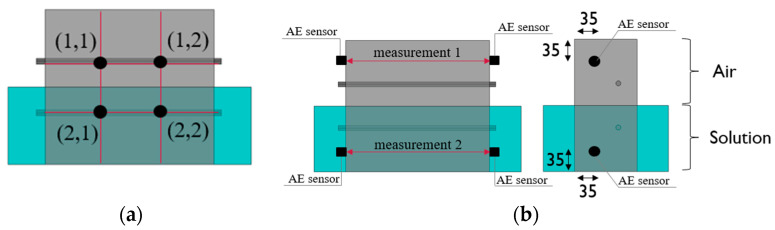
(**a**) Arrangement of measurement points using the Galva Pulse method and (**b**) the acoustic emission method (mm).

Reference measurements using the GalvaPulse method were made before immersing the specimens in the solution at a constant temperature, while measurements using the acoustic emission method were made before placing the specimens in the freezing chamber. These results were reference measurements for later measurements. The arrangement of the measurement points is shown in Figure 6a (GP method) and Figure 6b (AE method).

### 2.2. Research Methods

In order to assess the corrosion activity of the main reinforcement bars, the specimens were subjected to semi-destructive tests using the galvanostatic pulse method. To assess the impact of an aggressive environment on concrete and fiber-reinforced concrete, the non-destructive acoustic emission method was applied. In addition, tests accompanying the assessment of the compressive strength of the specimens were performed.

#### 2.2.1. Galvanostatic Pulse Method

**Figure 7:**
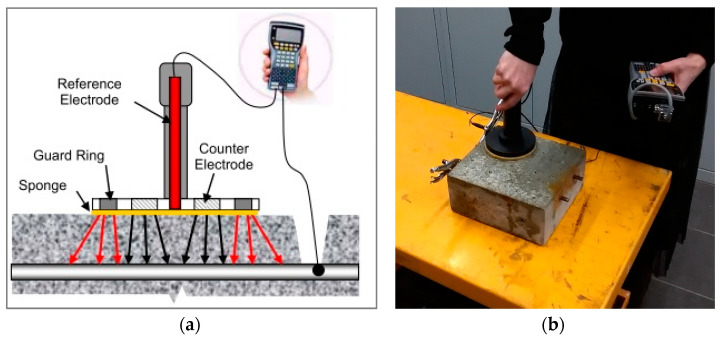
(**a**) Connection diagram of the GP-5000 GalvaPulse^TM^ set with the tested reinforcement bar [37]; (**b**) GP method measurement.

The galvanostatic pulse method belongs to the electrochemical polarization research methods group—the essence of its application results from the electrochemical process of corrosion of reinforcement in concrete. Concrete has a porous structure with pores filled with liquid, so it is a kind of electrolyte. The steel rebar is an electrode placed in an electrolyte. As a result of differences in the concentration of ions in the electrolyte and possible micro-defects in the steel, local anode and cathode areas are formed on the surface of the rod—microcells that initiate the flow of electrons—and the liquid-filled concrete is a carrier of ions. When such a cell is connected to a device equipped with an electric meter and a reference electrode, and the current flow is induced correctly, it is possible to obtain data on corrosion probability and estimate the corrosion activity. The GP-5000 GalvaPulse^TM^ measuring set was used in the described studies. It enables determining the probability of corrosion based on measurements of the stationary potential of reinforcement and resistivity of the concrete cover, estimating the reinforcement corrosion activity and predicting corrosion rate based on the measurements of the corrosion current density. The scheme of operation of the device in connection with a reinforced concrete element is shown in Figure 7a. Figure 7b shows the measurement with the GalvaPulse apparatus on a selected specimen.

Of the three parameters measured with the GP-5000 GalvaPulse^TM^ device, the most effective and reliable in the case of the presented tests were the measurements of the corrosion current density. In the case of the other two parameters, the obtained results could lead to their incorrect interpretation. This resulted from the influence of steel fibers, disturbing the measurements of the stationary potential of reinforcement [36], and from the young age of the specimens, which affects the measurements of the concrete cover resistivity [16]. Therefore, the analysis of the results obtained by the galvanostatic pulse method was based on the measurements of the corrosion current density. The obtained results were related to the borderline results presented in Table 2.

Corrosion current density (i_cor_) measurements were performed for three series of reinforced concrete specimens (C, SF_0.25 and SF_0.5) in two stages. The first stage included measurements on specimens before they were immersed and frozen in a 3% NaCl solution. The second stage concerned the measurements of specimens after 100 cycles of freezing and thawing in a solution. Measurements were made on the surface of each specimen at four measurement points located on the line of the reinforcement bar (Figure 5b), where the points with coordinates (1, 1) and (1, 2) concerned the bar not immersed in the solution, and the points with coordinates (2, 1) and (2, 2) concerned the immersed rod.

#### 2.2.2. Acoustic Emission Method

The acoustic emission method is one of the non-destructive methods, an overview of which can be found, among others, in [40,41,42]. It analyzes elastic waves propagated due to processes occurring inside and outside the material. Recommendations regarding damage detection and their assessment using the acoustic emission method can be found, for example, in [42,43,44,45]. A standard research direction is determining whether active destructive processes occur in the specimen. To determine this, during the test, the measuring apparatus registers the elastic waves propagating in the material as a result of the damage. Then, a team of specialists interprets and evaluates the results [46,47] and classes the damage [48,49]. One of the acoustic methods (IADP) consists of classifying the received acoustic emission signals based on assigning them to typical damage of the material, which is contained in the database of reference signals created before the test in the laboratory condition. Examples of the application of this method are given, for example, in [50,51], where it was used to identify destructive processes in the diagnosis of reinforced concrete objects. In [52,53,54], it was used to identify active destructive processes in unloaded concrete or in [55] for monitoring the course of the alkali-silica reaction. The difficulty of this method involves not only the need to develop a signal base of model destructive processes before testing but also to determine the velocity of the longitudinal wave, which is necessary to locate these processes. Analysis of elastic waves is the basis of acoustic methods, and study of the wave propagation in various materials is essential to best recognize the phenomena occurring in them. One of the directions of analysis is the characteristic of elastic wave propagation in parameters such as longitudinal wave velocity, amplitude, energy, frequency, duration, rise time or waveform. To perform a reliable analysis, the elastic wave is excited multiple times by an artificial acoustic emission source. Various methods of generating an elastic wave source exist, i.e., breaking a glass capillary [56,57], dropping a steel ball [56,58], breaking the graphite of a pencil in the Hsu Nielsen test [59,60], a simulated acoustic emission wave by AE system [61,62] or an ultrasonic wave by a pulse generator [63]. The artificial sources of acoustic emission are characterized by a short duration of the elastic wave, which is typical for a natural source of acoustic emission [64]. In the study of acoustic emission wave parameters in concrete, two methods of wave generating have been used in practice, i.e., breaking graphite in the Hsu Nielsen test [65] and a pulse from an acoustic emission sensor [66], which is currently not a very recognized source, probably due to the availability of the sensors.

**Figure 8:**
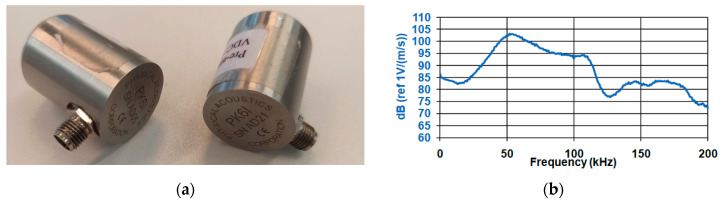
(**a**) Resonance sensor—PK6I, (**b**) graph of the sensitivity of the PK6I resonant sensor depending on the frequency [61].

In this paper, the repetitive source from the acoustic emission sensor was chosen to analyze the wave velocity and waveform. The advantage of this method of generating an acoustic wave is the reduction of the impact of human error. The test was performed using the PK6I sensor [61] with a resonant frequency of 55 kHz (Figure 8) with a built-in 26 dB preamplifier, operating in the frequency range of 35–65 kHz. In addition, the Pocket AE device with the built-in Auto Sensor Test system was used for the AE wave measurement. It made it possible to send an artificial wave of acoustic emission to the medium and simultaneously receive a wave from the medium [67]. 

**Figure 9:**
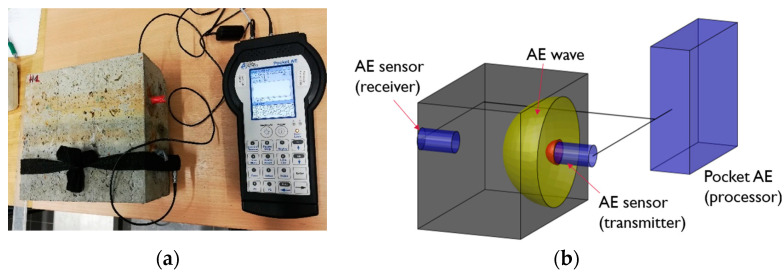
(**a**) Measuring stand with apparatus—Pocket AE; (**b**) diagram of AE wave propagation in a specimen.

Before starting the test, the sensor application site was adequately prepared by: cleaning and applying a coupling agent. In addition, sensors were calibrated using the Hsu-Nielsen test [59,60]. The acoustic emission test consisted in recording the arrival time of the longitudinal wave generated by the calibration pulse from the sensor. The generated elastic wave passed through one of the two tested zones of a given specimen (air or solution) (Figure 5a). In order to verify the errors of the apparatus, the wave was generated ten times by both sensors in two directions, while in order to eliminate overlapping of the waves, 10-second excitation intervals were adopted. The distance between the sensors was 228 mm. The location of the sensors on the specimen and the measuring stand are shown in Figure 9a. The measurement results obtained were analyzed using the Vallen System GmbH software.

#### 2.2.3. Compressive Strength Tests

**Figure 10:**
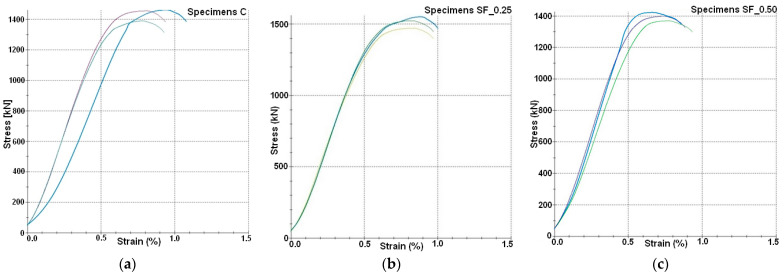
Stress-strain results measured in specimens: (**a**) C, (**b**) SF_0.25, (**c**) SF_0.50.

The tests of the specimens to determine the compressive strength were carried out 28 days after concreting following the standard recommendation [38,39]. Cubic specimens of 150 × 150 × 150 mm^3^ were destroyed in the SP-Z6000 Zwick/Roell tester with a maximum compressive force of 6000 kN. The specimens were continuously loaded to failure at a load speed of ~0.5 MPa/s. The results (Table 3), together with the graphs (Figure 10), were generated in the testXpert program compatible with the testing machine.

## 3. Research Results and Analysis

### 3.1. Analysis of Results Obtained from Tests Using the Galvanostatic Pulse Method

**Figure 11:**
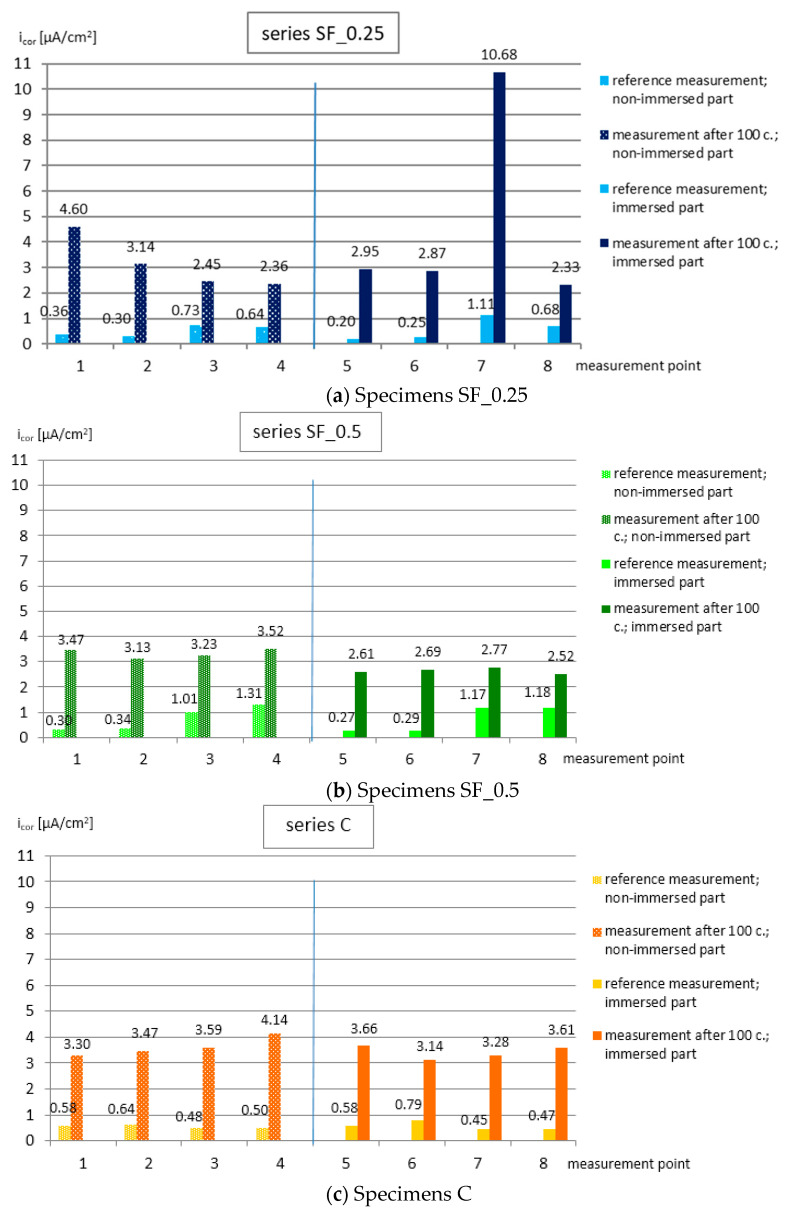
Corrosion current density values presented for individual series of specimens (**a**–**c**), respectively, for non-immersed parts (points 1–4) and immersed parts (points 5–8) in two measurement stages.

Figure 11 graphically presents the values of the corrosion current density obtained after two stages of measurements made on specimens of individual series (Figure 10a–c) for non-immersed parts (points 1–4) and immersed parts (points 5–8), respectively. In order to analyze the obtained results, they were compared to the data given in Table 2.

As seen from the graphs, the reference measurements (stage I) were in the range of i_cor_ = 0.2 ÷ 1.31 µA/cm^2^, which indicated unpredicted or irrelevant corrosion activity of the tested bars (Table 2). They proved no risk of corrosion. The values measured after 100 cycles of freezing and thawing specimens in 3% NaCl solution (stage II) at 23 measurement points were in the i_cor_ range of 2.33 ÷ 4.60 µA/cm^2^ (indicating low corrosion activity of the tested bars), and at one point, the current density increased to i_cor_ = 10.68 µA/cm^2^ (moderate corrosion activity of the tested rod). In all tested specimens, the corrosion activity of the reinforcement bars increased due to the action of chloride ions and cyclic freezing and thawing of the specimens.

In addition, Table 4 summarizes the results of the corrosion current density measured in the second stage of measurements for the specimens from individual series, separately for non-immersed and immersed parts. The table also includes the average values determined for the measurement points for specimens of the same series stored in the same ambient conditions (air or solution). In the case of the SF_0.25 series, due to one result that was significantly different from the others (* point 7: i_cor_ = 10.68 µA/cm^2^), the average of the results was presented in two variants: including this result and omitting it (values in parentheses).

Analysis of the results obtained from the tests (excluding the result of the SF_0.25 point 7 series) indicates that the corrosion activity of the main reinforcement bars after 100 cycles of freezing and thawing in 3% NaCl solution was the highest in the specimens of series C (concrete without fibers), with the highest increase in the average corrosion current density i_cor_ = 3.63 µA/cm^2^ (for non-immersed parts) and i_cor_ = 3.42 µA/cm^2^ (for immersed parts). Lower corrosion current density values were recorded in the specimens with the addition of fibers. In the specimens of the SF_0.5 series, the average values of the current density were i_cor_ = 3.34 µA/cm^2^ (for non-immersed parts) and i_cor_ = 2.65 µA/cm^2^ (for immersed parts), and the specimens of the SF_0.25 series (excluding the result of the SF_0.25-point series 7) were, respectively, i_cor_ = 3.14 µA/cm^2^ and i_cor_ = 2.72 µA/cm^2^. However, considering the value of the corrosion current density at point 7 in the SF_0.25 series specimen (i_cor_ = 10.68 µA/cm^2^), it should be assumed that a corrosion center was formed in the tested area. It could have resulted from micro-defects in the concrete cover (cracks caused by stresses in the concrete), which led to increased diffusion of Cl^−^ ions.

Analysis of the values of standard deviation and coefficient of variation (Table 4) shows that the dispersion of results was the smallest in the specimens of the SF_0.5 series. It follows that the addition of steel fibers in the amount of 0.5% of the volume of the concrete mix improved the coherence and tightness of the concrete cover. However, the greatest dispersion of results occurred in the specimens of the SF_0.25 series, in which both the lowest values of the corrosion current density (at most measurement points) and the highest values (at two measurement points) were recorded. It can therefore be assumed that the addition of fibers in the amount of 0.25% of the concrete mix volume was insufficient to improve the cohesion and tightness of the concrete cover comprehensively. Probably, the fibers locally limited the shrinkage and partially blocked the network of interconnected pores. However, due to their insufficient number, there were areas without fibers, where internal stresses in the concrete (caused initially by shrinkage and cyclic freezing and thawing of specimens) led to the formation of internal microcracks and thus more intensive diffusion of Cl^−^ ions into the lagging. The percentage of micro-reinforcement fibers dosed to reduce shrinkage in concrete is, therefore, significant, taking into account the effectiveness of the cover in protecting the reinforcement against corrosion.

The measurements of the corrosion current density made it possible to determine the corrosion activity of the reinforcement and estimate the corrosion rate. For this purpose, Faraday’s law was used to estimate that the corrosion current density with the value of i_cor_ = 1 (µA/cm^2^) corresponds approximately to the depth of loss of the cross-section of the reinforcing bar equal to 11.6 (µm/year) [37]. The maximum values of the corrosion current density were adopted for the analysis as the most representative for each series of specimens. On this basis, it was estimated that in the specimens of the C series, the corrosion rate may be 48.02 µm/year, in the specimens of the SF_0.5 series, the least, i.e., 40.83 µm/year, and in the specimens of the SF_0.25 series, the highest, i.e., 123.89 µm/year.

It is also worth noting the differences in the corrosion current density values that occurred depending on whether the measurement points were located in the non-immersed (air) or immersed (solution) part of the specimens. In most of the obtained results, the corrosion current density was lower for some of the specimens immersed in the solution (in the specimens of the SF_0.5 series, it concerned all measurement points). Therefore, the bars immersed in the solution were characterized by lower corrosion activity than those above the solution. This is probably related to the essence of the corrosion process. Although the concentration of chloride ions in the solution was probably higher than in the “salt mist” (not measured in this study), oxygen was necessary to develop reinforcement corrosion, thereby making chloride ion migration in the concrete above the solution easier than in the immersed parts of the specimens, whose capillaries were filled with liquid [20,21].

### 3.2. Analysis of Test Results Using the AE Method

**Figure 12:**
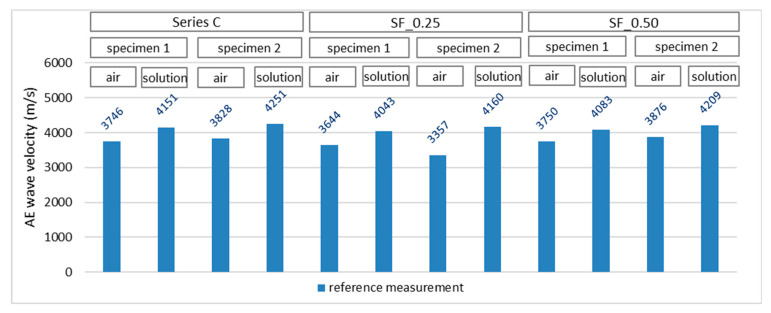
AE wave velocity values recorded in specimens—reference measurement.

The reference measurement using the acoustic emission method was made before the freeze–thaw cycles in the chamber, i.e., after 50 days of specimen care in a 3% NaCl solution. Measurements were made in two specimen zones (Figure 6b). The first measurement was made in the zone where the concrete cured in the air and the second in the part of the specimen previously immersed in the solution. AE signals in a given direction were generated alternately from each sensor. This means that after a series of ten excitations of the wave from sensor no. 1 and recording these waves by sensor no. 2., the order was reversed, and the wave was generated from sensor no. 2 and recorded with sensor no. 1. This procedure was aimed at verifying the obtained AE results by eliminating the situation related to the error resulting from the sensor’s defect. As a result, each sensor obtained a coefficient of variation not exceeding 0.5% in the values of the wave velocities generated independently. Figure 12 presents, in a graphical form, the average velocity values (reference for further measurements) of the propagating acoustic wave in individual specimens in stage I of the test.

The recorded wave parameters show that each immersed part of the specimens (100% care) had higher wave velocity values compared with those recorded in the part of the specimen treated in the air. In both cases, the maintenance time was the same (50 days). Therefore, immersion of some of the specimens in the solution positively affected the course of the cement hydration process, which was complete in relation to the part of the specimens left in the air.

**Figure 13:**
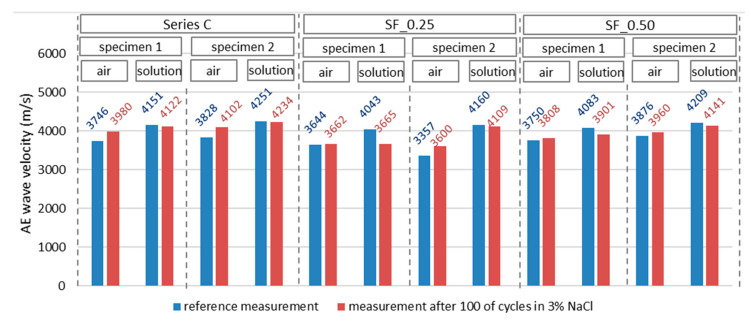
AE wave velocity values recorded in specimens in two measurement stages.

Figure 13 presents the values of the AE wave velocity propagating in individual specimens recorded during two stages of the test, with air or solution parts of the tested specimen taken into account. The first stage represents the measurement immediately after the curing time in the NaCl solution, while in the second stage, the specimen was subjected to 100 freeze–thaw cycles. The blue color indicates the results of reference measurements (stage I), and the red color indicates the results obtained after 100 freeze–thaw cycles in specimens immersed in 3% sodium chloride solution (stage II).

Table 5 summarizes the basic statistical parameters of the velocities obtained in concrete and fiber concrete tests. A low coefficient of variation distinguishes the research results.

**Figure 14:**
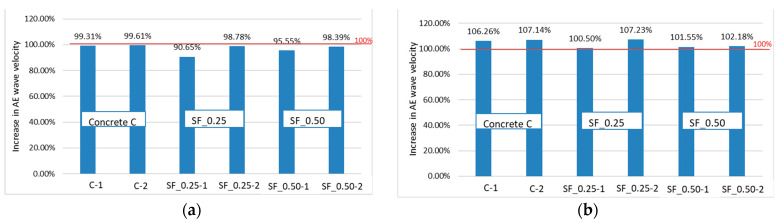
Increase in the velocity of the elastic wave recorded in the specimens (**a**) in the immersed part (measurement 2) and (**b**) in the non-immersed part (measurement 1).

Figure 14 shows the wave velocity increase after 100 cycles of heating and cooling, respectively, in some of the specimens immersed (Figure 14a) and those remaining in the air (Figure 14b) compared to the reference velocity (100%) before testing in the freezing chamber. In all specimens tested in the immersed part, a decrease in the AE wave velocity was observed, which indicates the negative impact of the heating and freezing cycles on the concrete structure in the part whose pores were filled with water. The highest decrease in wave velocity was observed in concrete SF_0.25 and amounted to 9.35%. It may indicate greater damage to the concrete in comparison to the remaining specimens.

In the second part of each specimen (non-immersed part), an increase in the velocity of the AE wave was observed, which may indicate the influence of humidity on the concrete sealing.

**Figure 15:**
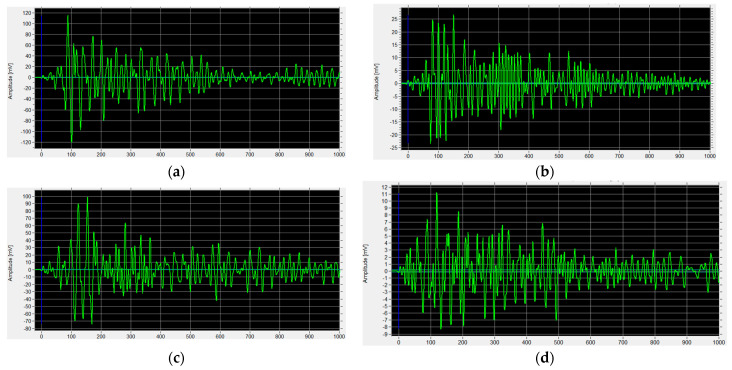
The waveform received by the sensor in stages I and II: (**a**) specimen SF_0.25—stage I (solution); (**b**) specimen SF_0.25—stage II (solution); (**c**) specimen SF_0.25—stage I (air); (**d**) specimen SF_0.25—stage II (air).

Figure 15 shows the waveform recorded by the AE sensor (receiver) during the test (amplitude vs. time) of the example specimen SF_0.25 at various stages of the test.

The waves recorded in the SF_0.25 in the second stage of the research, after 100 freeze-thaw cycles, had a lower amplitude than the amplitude recorded in the specimen in Stage I. In the immersed part, the amplitude decreased from 115 mV (Figure 15a) to approx. 25 mV (Figure 15b) and from 100 mV (Figure 15c) to 11 mV (Figure 15d) in the non-immersed part. The cause may include destructive processes in the fiber concrete resulting from freezing and thawing processes.

## 4. Discussion

Analysis of the test results shows that the corrosion activity of the main reinforcement bars after 100 cycles of freezing and thawing in 3% NaCl solution was the highest in the C series specimens (concrete without fibers), in which the average increase in corrosion current density was highest, icor = 3.63 µA/cm^2^.

The decrease in the wave velocity in the immersed parts during the freezing cycles was most likely due to micro-defect formation in the concrete. However, the bars in these parts of the specimens showed slightly lower reinforcement corrosion activity than the bars in the non-immersed parts of the specimens. It can therefore be assumed that, despite greater losses in the concrete cover, the ambient humidity of 100% contributed to the slowdown of the corrosion process, probably due to the oxygen deficit, which is necessary for the process to proceed.

In the non-immersed parts of the specimens, there was no such destruction of concrete resulting from the freezing and thawing cycles. The effect of filling the voids of the concrete pores with corrosion products resulted in the “sealing” of the concrete and increasing the wave velocity. Hence, further tests are planned to supplement the AE wave measurements, the location and quantitative assessment of destructive processes taking place in unloaded concrete in an analogous environment in order to demonstrate the cause of the increased number of destructive processes in the submerged zone compared to the non-immersed zone.

**Figure 16:**
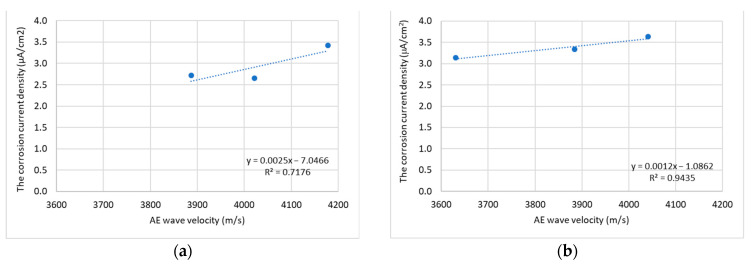
Trend lines between the corrosion current density and AE wave velocity of specimen: (**a**) for the immersed part of the specimens; (**b**) for the non-immersed part of the specimens.

The results of the GP and AE tests showed a strong relationship between the tested parameters, i.e., the corrosion current density and AE wave velocity. The Pearson coefficient determined for these parameters for the immersed part of the specimens is 0.85, while in the non-immersed part it is 0.97. This information is important in the context of the possibility of developing a non-destructive method that enables the diagnosis of corrosion in reinforced concrete and fiber-reinforced concrete based on the analysis of the AE wave velocity. Figure 16 shows the correlation between the mentioned parameters, the value of R2 and the equations of the assumed linear functions.

Initially, it can be concluded that in concrete elements cured in water containing NaCl, the development of the corrosion process can be signaled based on wave velocity analysis. It was observed that, due to the possible filling of concrete voids with corrosion products, partial sealing of the concrete might occur, corresponding to a local increase in the wave velocity relative to places not exposed to corrosion development. However, this dependence requires further analysis and in-depth research.

In particular, the last issues related to the correlation of the longitudinal wave velocity with the degree of rod corrosion and the impact of concrete moisture on the longitudinal wave velocity (stage I) or the effect of cyclical heating and freezing of concrete on the longitudinal wave velocity (stage II) can be considered as a novelty in the conducted tests. However, to fully confirm the observed phenomena, the work in this direction should be continued due to the scope of work carried out so far.

For this reason, one of the directions of further work will be verifying the correlation between the wave velocity change and the progressive corrosion of reinforced concrete elements.

In addition, tests will be carried out to compare the method of sending a reference source in the form of the Hsu-Nielsen method (wave generated by breaking a pencil lead) and from a sensor, determining the advantages and disadvantages of both methods of generating a longitudinal wave source and measuring its velocity for samples subjected to NaCl and cyclic heating and cooling.

These tests will also include comparisons of elastic wave shapes and amplitudes and the impact of the corrosive degradation of concrete on these parameters.

## 5. Conclusions

As a result of the conducted research and analysis, the following conclusions can be drawn:

1. The corrosion activity in the specimens with 0.5% fibers was the lowest, and the dispersion of the results was the smallest. The highest corrosion activity was shown by bars in the concrete specimen without fibers. The largest scatter of results was observed in the specimen with the addition of 0.25% of fibers. This indicates that the addition of steel micro-reinforcement fibers to concrete affects the effectiveness of the cover as a layer protecting the reinforcement against corrosion caused by the action of chloride ions and frost. However, the percentage of fiber content in the concrete mixture is of significant importance.
2. The content of steel fibers in the concrete mixture in the amount of 0.25%, defined as the minimum anti-shrinkage micro-reinforcement in concrete, is insufficient to obtain a homogeneous and tight concrete cover protecting the reinforcement against corrosion.
3. The use of steel fibers as micro-reinforcement does not increase the corrosion risk of the main reinforcement in concrete.
4. Randomly dispersed fine steel fibers covered with concrete do not constitute corrosion centers.
5. Corrosion caused by chloride ions is pitting corrosion, which means that in concrete elements, there may be point corrosion centers with high corrosion activity of the reinforcement.
6. Heating and freezing cycles in a 3% NaCl water solution affect the destruction of concrete—the wave velocity and amplitude decreased in this medium.
7. There is a strong linear correlation between the AE wave velocity induced by the calibration pulse from the PK6I acoustic sensor and the values of the corrosion current density recorded in the main reinforcement bars.

Based on the results and presented conclusions, it can be predicted that further tests will allow for the determination of the procedure for inferring the reinforcement corrosion activity based on the AE tests and their correlation to the GP tests.

## Figures and Tables

**Table 1:** The compositions of concrete mixture.

Ingredients	Quantity per 1 m^3^ of Concrete Mixture
Portland cement CEM I (42,5 N-MSR/NA)	384 kg
Mine sand	680 kg
Basalt aggregate 2 ÷ 8	600 kg
Basalt aggregate 8 ÷ 16	650 kg
Water	166 L
Plasticizer ADVA Flow 440 (BV/FM)	0.5% (per 1 kg of cement)
Air entrainer Darex AEA W (LP)	0.2% (per 1 kg of cement)

**Table 2:** Criteria for assessing the degree of reinforcement corrosion risk resulting from measurements of the corrosion current density carried out using the galvanostatic pulse method.

	Reinforcement Corrosion Activity, i_cor_ (μA/cm^2^)		Forecasted Rate of Corrosion
Corrosion Current Density	<0.5	Not forecasted	<0.006 mm⋅year^−1^
	0.5 ÷ 2.0	Irrelevant	0.006 ÷ 0.023 mm⋅year^−1^
	2.0 ÷ 5.0	Low	0.023 ÷ 0.058 mm⋅year^−1^
	5.0 ÷ 15.0	Moderate	0.058 ÷ 0.174 mm⋅year^−1^
	>15.0	High	>0.174 mm⋅year^−1^

**Table 3:** The results of the compressive strength of the tested concrete and fiber-reinforced concrete after 28 days of curing.

Specimens	C	SF_0.25	SF_0.50
1	64.93	65.41	63.19
2	64.71	68.91	60.88
3	61.74	67.71	62.16
Mean value	63.79	67.34	62.08
Stand. dev.	1.45	1.45	0.94
Variation (%)	2.28	2.16	1.52

**Table 4:** Corrosion current density values determined in stage II of measurements for individual series of specimens (non-immersed and immersed parts).

	Specimens SF_0.25		Specimens SF_0.50		Specimens C	
	Air	Solution	Air	Solution	Air	Solution
Stage II	4.60	2.95	3.47	2.61	3.30	3.66
	3.14	2.87	3.13	2.69	3.47	3.14
	2.45	10.68 *	3.23	2.77	3.59	3.28
	2.36	2.33	3.52	2.52	4.14	3.61
Mean value (MPa)	3.14	4.71 (2.72)	3.34	2.65	3.63	3.42
Standard deviation (MPa)	0.90	3.46 (0.28)	0.16	0.09	0.31	0.22
Coefficient of variation (%)	29	70 (10)	5	4	9	6

**Table 5:** Acoustic wave velocity values recorded in specimens C, SF_0.25 and SF_0.50 with statistical parameters.

	AE Wave Velocity (m/s)					
	Series C		Series SF_0.25		Series SF_0.50	
	Air	Solution	Air	Solution	Air	Solution
	Stage I					
Specimen 1	3746	4151	3644	4043	3750	4083
Specimen 2	3828	4251	3357	4160	3876	4209
Mean value (m/s)	3787	4201	3501	4101	3813	4146
Stand. dev. (m/s)	41.3	49.9	143.1	58.5	62.9	63.1
Coeff. of variation (%)	1.1	0.01	0.04	0.01	0.02	0.02
	Stage II					
Specimen 1	3980	4122	3662	3665	3808	3901
Specimen 2	4102	4234	3600	4109	3960	4141
Mean value (m/s)	4041	4178	3631	3887	3884	4021
Stand. dev. (m/s)	60.7	55.9	30.8	221.9	76.1	120.1
Coeff. of variation (%)	0.02	0.01	0.01	0.06	0.02	0.03

## Data Availability

Not applicable.
